# Blood pressure variability and mortality in patients admitted with acute stroke in a tertiary care stroke centre (2016–2019): a retrospective cohort study

**DOI:** 10.1136/bmjopen-2024-095773

**Published:** 2025-05-15

**Authors:** Mohamed Tawengi, Rizeq F Hourani, Tamader Alyaarabi, Ahmed Adel Elsabagh, Yazan Al-Dali, Rama Ghassan Hommos, Jawaher Baraka, Abdelaziz M Tawengi, Bushra M Abdallah, Ahmad Hatem, Sundus Sardar, Yahia Z Imam, Naveed Akhtar, Muhammad Zahid, Suhail Doi, Mohammed Ibn-Masud Danjuma, Abdelnaser Elzouki

**Affiliations:** 1 Internal Medicine Residency Program, Division of General Medicine, Hamad Medical Corporation, Doha, Qatar; 2 College of Medicine, Qatar University, Doha, Qatar; 3 Diagnostic Radiology Residency Program, Division of Clinical Imaging, Hamad Medical Corporation, Doha, Qatar; 4 Definition of General Surgery, Department of Surgery, Hamad Medical Corporation, Doha, Qatar; 5 Urology Department, Hamad Medical Corporation, Doha, Qatar; 6 Department of Medicine, Penn State University, Milton S Hershey Medical Center, Hershey, Pennsylvania, USA; 7 Harvard T.H. Chan School of Public Health, Boston, Massachusetts, USA; 8 Neuroscience Institute, Hamad Medical Corporation, Doha, Qatar; 9 Weill Cornell Medical College in Qatar, Doha, Qatar; 10 Health Sciences Centre, University of Manitoba, Winnipeg, Manitoba, Canada; 11 Department of Internal Medicine, Hamad Medical Corporation, Doha, Qatar; 12 Department of Population Medicine, Qatar University, Doha, Qatar

**Keywords:** Stroke, Mortality, Blood Pressure

## Abstract

**Abstract:**

**Objectives:**

The influence of short-term variations in blood pressure (BP) in acute stroke on clinical outcomes remains uncertain. Our study explores the relationship between BP variability (BPV) from stroke admission up to 72 hours and in-hospital and 1-year mortality.

**Design:**

Retrospective observational cohort study.

**Setting:**

Hamad General Hospital (HGH) a tertiary care stroke centre in Qatar.

**Participants:**

2820 participants were initially included. After the exclusion of ineligible subjects, 2554 patients (82.5% male, median age 53±9 years) were included. 893 (34.96%) were from the Middle East and North Africa, 1302 (50.98%) were from South Asia, 258 (10.10%) from Southeast Asia, 9 (0.35%) were from East Asia and 92 (3.60%) were from other regions. Eligible participants were adult patients above 18 years of age who presented with acute ischaemic or haemorrhagic stroke. Excluded individuals were those younger than 18 years, had incomplete data, had transient ischaemic attack (TIA), had severe hypoglycaemia on admission (<3.3 mmol/L) or had a history of chronic kidney disease (CKD).

**Interventions:**

We measured the BP every 4 hours over 3 days with a total of 18 readings from stroke admission. We then categorised BPV into five (L1–L5) and four (L1–L4) levels for systolic and diastolic BPs, respectively, and evaluated their association with mortality.

**Results:**

There were increased odds of in-hospital mortality with increased systolic and diastolic variability (L2, OR 2.64, 95% CI 1.44 to 4.84; L3, OR 4.20 95% CI 2.14 to 8.24; L4, OR 10.14, 95% CI 4.93 to 20.85; L5, OR 23.18, 95%CI 10.88 to 49.37), (p=0.002 to <0.001) and (L2, OR 1.61, 95% CI 0.96 to 2.69; L3, OR 2.95, 95% CI 1.70 to 5.12 and L4, OR 8.00, 95% CI 4.49 to 14.25), (p=0.071 to <0.001), respectively. This was consistent with 1-year mortality for systolic and diastolic BPs.

**Conclusion:**

In a retrospective cohort of ethnically diverse acute stroke patient population, BPV was significantly associated with both in-hospital and 1-year mortality. Further prospective research is needed to define BPV and establish interventions and management accordingly.

**STRENGTHS AND LIMITATIONS OF THIS STUDY**

- Our study included 2554 patients which is a large cohort that adds to the validity and generalisability of the results.
- A minimum of 10 blood pressure (BP) readings over 72 hours was required for inclusion, giving a solid view of the impact of BP variability (BPV) on the outcomes of stroke.
- The follow-up period of 1 year with inverse probability weighted logistic regression provides a robust insight into the long-term consequences of BPV on the outcomes of stroke.
- Our study is limited by its retrospective design, missing values and absence of consistent synchrony in the timing of BP measurements by nursing personnel.
- The stability of point estimates of our study outcomes meant that these limitations did not significantly confound our findings.

## Introduction

 High blood pressure (BP) is independently associated with poor outcomes in acute stroke.^1 2^ Additional haemodynamic parameters including systolic BP (SBP), diastolic BP (DBP), mean arterial pressure (MAP), pulse pressure and heart rate have been associated with poor outcomes following stroke.^3^ Evidence investigating acute BP variability (BPV) as a determinant of stroke outcomes is increasing.^4^,^9^ However, no clear definition of BPV is yet defined by the guidelines. Systolic BPV (SBPV) is being recognised as an important triggering factor for vascular events including stroke and cardiovascular events.^10^ Likewise, some authors suggest that diastolic BPV (DBPV) may be as important as SBPV.^11 12^ It is, however, unclear whether the control of acute SBPV and DBPV after acute stroke would provide a potentially modifiable therapeutic target and improved clinical outcomes in patients with acute stroke.

Evidence on the effect of BPV after acute stroke on the clinical outcomes remains limited. A post hoc analysis of the Intensive BP Reduction in Acute Cerebral Hemorrhage Trial 2 (INTERACT2) dataset reported significant associations between SBPV in the hyperacute (first 24 hours) and acute (days 2–7) periods, death and disability at 90 days in 2839 participants with acute intracerebral haemorrhage (ICH) (<6 hours of symptom onset) and elevated SBP (SBP >150 mm Hg).^5^ Other studies have mostly assessed BPV during a period of ≥24 hours; the majority found significant associations between SBPV or DBPV and poor long-term functional outcome (≥3 months),^13^,^17^ or adverse findings on repeat neuroimaging,^14 18^ although not all.^19 20^ Two small studies have examined the effect of short-term BPV on outcomes in acute ischaemic stroke using beat-to-beat BP monitoring. Dawson *et al* evaluated the effect of BPV in patients with ischaemic stroke and reported that DBPV and MAP variability predict a poor 30-day outcome,^21^ but Graff *et al* found no BPV difference between good and poor outcome groups at 90 days.^22^

To date, no study has assessed the effect of short-term SBPV and DBPV derived from casual cuff BP measures –the most commonly used BP monitoring index for patients with acute stroke. The recommendations on this method of BP measurement are provided by the American Heart Association.^23^ BP measurement is done by trained nurses mostly, using a standard mercury sphygmomanometer and the appropriate cuff size based on the arm circumferences with the patient in supine position. Knowledge on how best to measure and define BPV has not been explored. Additionally, available evidence on the effect of BPV on outcome after acute stroke in our region is scarce.

Our observational study explores the relationship between BPV, measured every 4 hours over 3 days with a total of 18 readings from stroke admission and derived from multiple closely spaced casual BP measures, and in-hospital and 1-year mortality.

## Methods

### Study design and participants

This is a retrospective cohort study of patients presenting with acute cerebrovascular accidents (ischaemic stroke or haemorrhagic stroke) to Hamad General Hospital (HGH) between 1 May 2016 and 30 June 2019. HGH, a member of Hamad Medical Corporation (HMC), is regarded as the central hub for acute stroke care in Qatar. Over 80% of stroke cases are admitted or referred to HGH, thereby potentially reflecting acute stroke care in the whole of Qatar.^24^ A multiethnic stroke database was established at HGH in January 2014, where clinical details were systematically recorded by trained individuals, including the clinical presentation, severity of deficits and National Institutes of Health Stroke Scale (NIHSS), TOAST classification, risk factors, complications and outcome of all patients admitted with stroke, among other variables. All eligible individuals during the predetermined timeframe were recruited from the database for this study.

Eligible participants were all adult patients above the age of 18 years who presented to HGH with an acute ischaemic stroke or haemorrhagic stroke. Excluded individuals were those younger than 18 years, had incomplete data regarding the diagnosis or outcomes, had transient ischaemic attack (TIA), had severe hypoglycaemia on admission (<3.3 mmol/L) or had a history of chronic kidney disease (CKD).

### Patient involvement

Considering the retrospective design of the study and the use of the aforementioned stroke database as the source of our data, patients’ involvement in the conduction of this research was not possible.

### Data collection and variables

Data collection on the included participants was obtained from the stroke database and supplemented with the patients’ electronic medical records. Collected data were then entered into a standardised data collection sheet on Microsoft Excel.

Data were obtained on the following: (1) baseline demographics including age, sex, nationality and medical comorbidities; (2) stroke type, whether ischaemic stroke, TIA or haemorrhagic stroke, and the TOAST classification; (3) severity of stroke based on NIHSS scale; (4) BP readings on presentation and during the following 72 hours recorded every 4 hours with six readings per day, obtained using standard Dinamap, which is utilised across the hospital and (5) mortality, including in-hospital and mortality 1 year post stroke.

The SD of SBPV was then categorised for each person into five levels; (L1, <11 mm Hg; L2, 12–16 mm Hg; L3, 17–21 mm Hg; L4, 22–26 mm Hg; L5, >27 mm Hg). For DBPV, patients were grouped into four levels as follows: (L1, <8 mm Hg; L2, 9–11 mm Hg; L3, 12–15 mm Hg; L4, >16 mm Hg). Patients with 8 or more missing values out of 18 were excluded, and the SDs were computed for those who had 10 or more readings over 3 days. These levels of SD were utilised as the measure of SBPV or DBPV over the first 3 days of admission. These two variables were used as categories in lieu of the continuous measure to avoid problems with non-linearity. Additionally, we calculated the flux of patients (the difference between the highest and the lowest BP readings) for both systolic and diastolic BPs as a clinical representation of variability. It was then tabulated by the SD categories, and the median values (p50) were reported.

For the purpose of this study, ischaemic stroke, TIAs and intracranial haemorrhage (ICH) were diagnosed according to the WHO criteria. Mortality was confirmed by reviewing the death note in patients’ medical records for both in-hospital and 1 year post-stroke mortality.

### Statistical analysis

Descriptive statistics of the cohort data were presented as medians and interquartile ranges. Wilcoxon rank sum test was employed to compare the differences between groups. For categorical variables, frequencies and percentages were reported and compared using Pearson’s χ^2^ test.

To investigate the association of BPV with in-hospital mortality, we used multivariable regression. Adjustments were made for variables that were either potentially confounding or prognostic for the outcome based on a directed acyclic graph (DAG). These covariates included type of stroke (ischaemic vs haemorrhagic), age, hypertension (history or newly diagnosed) and history of cardiac disease. The analysis was repeated for assessment of the impact of BPV on 1-year mortality.

As we had significant losses to complete follow-up within the 1-year regression model, we adjusted for the dropouts from the model by running an appropriately weighted regression. We first computed the probability to remain in the study at 1 year by regressing (using logistic regression) an indicator variable on several baseline explanatory variables. The fitted model gave a predicted probability for each person that someone with those characteristics would be in the model at 1 year. Each person was then given a weight equal to 1 /p, where p is their fitted probability of being in the final logistic regression model. The weighted analysis was reported, although it was not substantially different from the unweighted analysis. An inverse probability weighted logistic regression with a robust error variance was fitted to the data using a logit-link function and a binary response variable for mortality. By applying this model, the ORs and 95% CIs were obtained.

We used a p-value threshold of 0.05 to decide on rejection or not of the null hypothesis. The null hypothesis was defined as the mortality difference between groups defined by BPV was zero. Exact p-values and 95% CIs were reported for inference and to quantify precision respectively. All statistical analyses were carried out using Stata 18.

## Results

**Figure 1:**
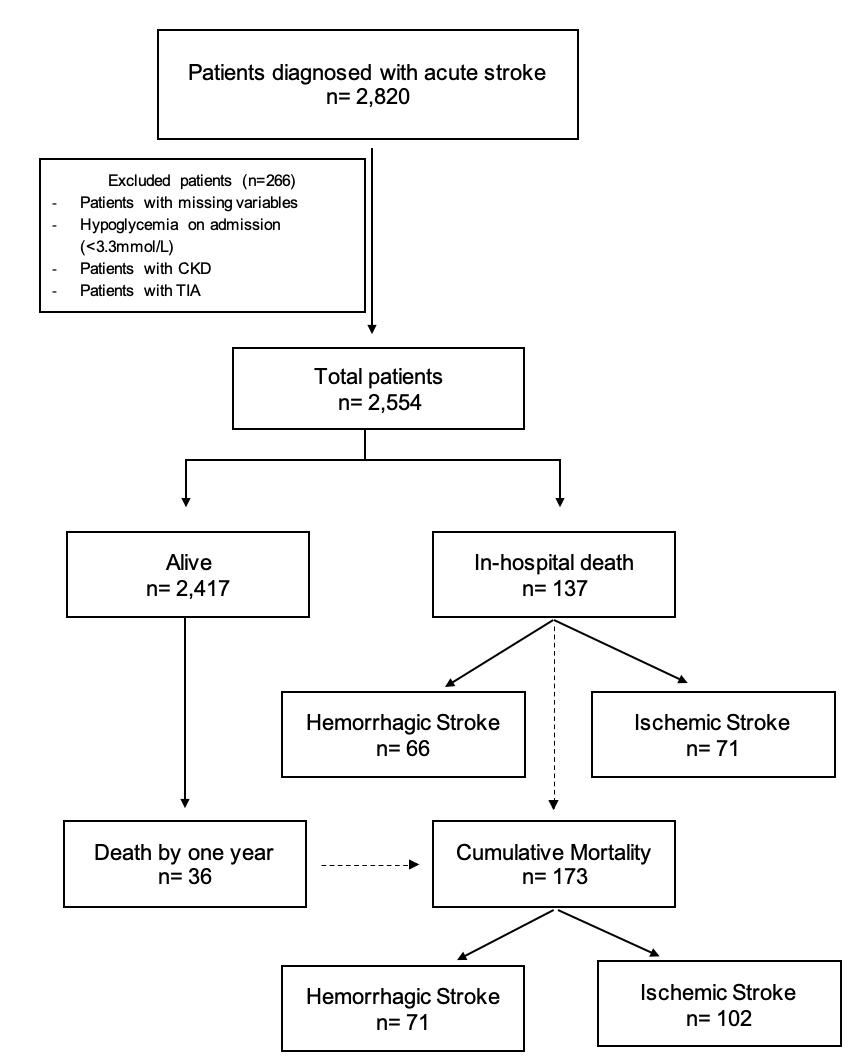
Study timeline. CKD, chronic kidney disease; TIA, transient ischaemic attack.

Figure 1 shows the flow chart of the study. After the exclusion of ineligible subjects, 2554 patients were available for analysis. 2417 (94.6%) patients survived while 137 (5.4%) died during admission. 36 additional patients died by 1-year follow-up, raising the total mortality at 1 year to 173/2,572 (6.7%).

Online supplemental table 1 shows the baseline characteristics. The median age for all participants was 53 years (44–62); 2108 (82.5%) were men and 446 (17.5%) were women. 893 (34.96%) were from the Middle East and North Africa (MENA), as defined by the International Monetary Fund.^25^ 1312 (51.01%) were from South Asia, 261 (10.15%) from Southeast Asia, nine (0.35%) were from East Asia and 93 (3.62%) were from other regions as defined by the United Nations' geoscheme which divides Asia into East, South and Southeast Asia.^26^ 2133 (83.5%) were diagnosed with ischaemic stroke while 421 (16.5%) had ICH. Patients who died were more likely to be men and older in age. Detailed baseline characteristics including NIHSS score on admission, comorbidities and laboratory investigations’ results on admission are presented in online supplemental table 1).

### Association between SBPV and in-hospital mortality

An adjusted multivariable logistic regression model (table 1) showed an increase in mortality odds as SBPV level increased over level 1 (L2, OR 2.64, 95%CI 1.44 to 4.84; L3, OR 4.20, 95%CI 2.14 to 8.24; L4, OR 10.14, 95%CI 4.93 to 20.85 and L5, OR 23.18, 95%CI 10.88 to 49.37). The p values for these ORs suggested that the null effect model was unlikely to have generated the study data (p=0.002 to <0.001). The model had adequate goodness of fit (AUC=0.80) and goodness of link as determined by a link test in Stata.

**Table 1:** Association between SBPV and in-hospital mortality—adjusted multivariable logistic regression model

Level of SBPV (p50)	OR*	95% CI	P value
<11 mm Hg (34 mm Hg)	1		
12–16 mm Hg (50 mm Hg)	2.64	1.44 to 4.84	0.002
17–21 mm Hg (68 mm Hg)	4.20	2.14 to 8.24	<0.001
22–26 mm Hg (89 mm Hg)	10.14	4.93 to 20.85	<0.001
>27 mm Hg (117 mm Hg)	23.18	10.88 to 49.37	<0.001

* Adjusted for type of stroke (ischaemic vs haemorrhagic), age, hypertension (history or newly diagnosed) and history of cardiac disease.

SBPV, systolic blood pressure variability.

### Association between DBPV and in-hospital mortality

An adjusted multivariable logistic regression model (table 2) showed an increase in mortality odds as DBPV level increased over level 1 (L2, OR 1.61, 95%CI 0.96 to 2.69; L3, OR 2.95, 95%CI 1.70 to 5.12 and L4, OR 8.00, 95%CI 4.49 to 14.25). The P values for these ORs suggested that the null effect model was unlikely to have generated the study data (p=0.071 to <0.001). The model had adequate goodness of fit (AUC=0.79) and goodness of link as determined by a link test in Stata.

**Table 2:** Association between DBPV and in-hospital mortality – adjusted multivariable logistic regression model

Level of DBPV (p50)	OR*	95% CI	P Value
<8 mm Hg (25 mm Hg)	1		
9–11 mm Hg (37 mm Hg)	1.61	0.96 to 2.69	0.071
12–15 mm Hg (50 mm Hg)	2.95	1.70 to 5.12	<0.001
>16 mm Hg (70 mm Hg)	8.00	4.49 to 14.25	<0.001

* Adjusted for type of stroke (ischaemic vs haemorrhagic), age, hypertension (history or newly diagnosed) and history of cardiac disease.

DBPV, diastolic blood pressure variability.

### Association between SBPV and 1-year mortality

An inverse probability weighted logistic regression model (table 3) showed an increase in mortality odds as SBPV level increased over level 1 (L2, OR 2.30, 95%CI 1.31 to 4.02; L3, OR 3.51, 95%CI 1.86 to 6.61; L4, OR 8.60, 95%CI 4.33 to 17.08 and L5, OR 19.27, 95%CI 9.25 to 40.15). The p values for these ORs suggested that the null effect model was unlikely to have generated the study data (p=0.004 to <0.001).

**Table 3:** Association between SBPV and 1-year mortality – inverse probability weighted logistic regression model

Level of SBPV (p50)	OR*	95% CI	P Value
<11 mm Hg (34 mm Hg)	1		
12–16 mm Hg (50 mm Hg)	2.30	1.31 to 4.02	0.004
17–21 mm Hg (68 mm Hg)	3.51	1.86 to 6.61	<0.001
22–26 mm Hg (89 mm Hg)	8.60	4.33 to 17.08	<0.001
>27 mm Hg (117 mm Hg)	19.27	9.25 to 40.15	<0.001

* Adjusted for type of stroke (ischaemic vs haemorrhagic), age, hypertension (history or newly diagnosed) and history of cardiac disease.

SBPV, systolic blood pressure variability.

### Association between DBPV and 1-year mortality

An inverse probability weighted logistic regression model (table 4) showed an increase in mortality odds as DBPV level increased over level 1 (L2, OR 1.49, 95%CI 0.91 to 2.41; L3, OR 2.20, 95%CI 1.26 to 3.84 and L4, OR 6.33, 95%CI 3.55 to 11.28). The p values for these ORs suggested that the null effect model was unlikely to have generated the study data (p=0.11 to <0.001).

**Table 4:** Association between DBPV and 1-year mortality – inverse probability weighted logistic regression model

Level of DBPV (p50)	OR*	95% CI	P Value
<8 mm Hg (25 mm Hg)	1		
9–11 mm Hg (37 mm Hg)	1.49	0.91 to 2.41	0.11
12–15 mm Hg (50 mm Hg)	2.20	1.26 to 3.84	0.006
>16 mm Hg (70 mm Hg)	6.33	3.55 to 11.28	<0.001

* Adjusted for type of stroke (ischaemic vs haemorrhagic), age, hypertension (history or newly diagnosed) and history of cardiac disease.

DBPV, diastolic blood pressure variability.

## Discussion

Our study consistently demonstrated a strong relationship, indicating that elevated BPV is linked to in-hospital mortality and cumulative all-cause mortality at 1 year. This association holds true for both SBP and DBP, as observed through early casual in-hospital BP measurements in a multiethnic population mainly from Middle East, North Africa and South Asia. These findings are at variance with what is currently reported in literature. Additionally, even where limited data is available, there is a paucity of this with regards to DBPV on stroke outcomes. Thus far, there is a paucity of robust evidence to objectively guide recommendations on the frequency of BP measurements to achieve the best prognostic value of BP readings in terms of stroke-related in-hospital mortality and combined all-cause mortality. Furthermore, the lack of definition of critical BPV further complicates the situation, as there is no agreement on an acceptable range of variability vs a concerning one requiring intervention that is reported in guidelines for stroke management. Therefore, our examination of this large cohort of patients admitted with acute stroke has provided the first tranche of clinical association between BPV and mortality. This could hold clinical implications on the current practice, resulting in proper future definition of variability.

We found that an increase in SBPV above 11 mm Hg (level 1) as well as an increase in DBPV above 8 mm Hg (level 1) was significantly associated with increased in-hospital and 1-year mortality in patients presenting with acute ischaemic or haemorrhagic stroke. Our results are consistent with recent reports from diverse patient populations which assessed the impact of BP variation on stroke outcomes. In an observational study that utilised data on patients previously enrolled in the China Antihypertensive Trial in Acute Ischemic Stroke (CATIS) trial and exclusively carried out on Chinese patient cohorts,^27 28^ He *et al* reported that 25.20% of patients died or had major disability due to BPV within a follow-up period of 3 months, with patients with the highest systolic fluctuations having the highest risk for such outcomes.^29 30^ A similar result was also found for the association of DBP fluctuations with study outcomes.^28^ In one study which recruited patients presenting with mild stroke and large vessel occlusion undergoing best medical management (intravenous thrombolysis (IVT), anticoagulation and antiplatelet), it was concluded that early neurological deterioration, defined as an increase in NIHSS score of ≥ 4 points within 24 hours, was evident in the group with the highest admission SBP readings and SBPV.^31^ Another study done in 2021 in Japan concluded that increases in the coefficient of variance of SBP and DBP were significantly associated with an increased risk of recurrent stroke. Additionally, the coefficient of variance of SBP and DBP was significantly associated with an increased risk of all-cause death.^32^ With respect to ICH, an abstract investigating patients who survived ICH in a period between 60 and 120 days after discharge was supportive that BPV is an important determinant of mortality and functional outcome in ICH survivors.^29^ Moreover, in one prospective study, BPV in acute ischaemic stroke was found to be negatively associated with favourable functional outcomes at 3 months, in addition to an increase in infarct expansion and higher risk of haemorrhagic transformation.^30^

Our results, in addition to the results reported in the literature, can be explained through multiple proposed pathophysiological mechanisms that can lead to the deterioration of neurological status and worsening of stroke outcomes. To begin with, the potential of reverse causality between BPV and stroke outcome must be taken into consideration, as BPV may be an outcome of worsening neurological condition. During the acute phase of stroke, BP control is affected due to the involvement of the autonomic nervous system,^33^ with baroreceptor reflex dysregulation being proposed as one of the mechanisms leading to BPV due to alterations in control of vasomotor tone and a reduction in cardiac baroreceptor sensitivity, although the exact mechanism remains unclear.^34^

On the other hand, BPV exacerbates the condition of the tissue affected by stroke, leading to poorer neurological outcomes. The frequent rise in BP in haemorrhagic stroke can lead to the growth of the haematoma and an increase in the arterial bleeding, while a sudden drop in BP may be a promoting factor for perihaematomal ischaemia. Blood–brain barrier can also be disrupted because of this variability, causing vasogenic oedema.^35^ As for ischaemic stroke, the cerebral blood flow in the affected tissue becomes dependent on the systemic BP since the cerebral autoregulation mechanisms are impaired. This means an increase in the BP may lead to cerebral oedema or haemorrhagic transformation in the focus of infarct. Additionally, the drop in systemic pressure reduces flow to the penumbra, worsening its ischaemia and increasing the infarct size.^34^ Overall, dynamic cerebral autoregulation impairment can explain why increased BPV is connected to poor prognosis in terms of death and disability in acute stroke.^34^ Causes of death can be due to these changes in a tight pressure-sensitive compartment like the skull, which can lead to neurological death due to acute rise in intracranial pressure and herniation or from complications due to immobility such as infections.^36^

On the other hand, despite the fact that our results were strongly supportive of the association between the increase in SBVP and DBPV and worsening stroke outcomes, other studies have dismissed these results.^37^,^41^ A study investigated the influence of BPV on functional outcomes and the occurrence of ICH within the population enrolled in the BP TARGET trial. Their analysis revealed no significant association between BPV and either functional outcomes or the incidence of ICH. Another interesting finding is that BPV in this population was more likely in the group with strict SBP target and control.^37^ Another study conducted in 2020 found that although increased early SBPV was related to worse functional outcome when patients had been treated by endovascular therapy, there was no association found in patients treated by intravenous thrombolysis.^38^ In a paper published in 2018, even though they concluded that beat-to-beat BPV was a predictor for stroke recurrence, they found that short-term BPV on ambulatory BP monitoring was not associated with stroke recurrence or cardiovascular events in patients after a TIA or a non-disabling stroke.^39^ A way to improve BPV was suggested in a 2017 study in Russia which showed that anxiety-related BPV may be improved by use of an anxiolytic within the medications given to improve BP control.^40^ In the GOLIATH trial, which included patients who had endovascular therapy with general anaesthesia after an acute ischaemic stroke, no association was found between any BP parameter, including BPV and neurological outcome.^41^

Based on the meta-analysis done in 2023, there is still not enough consensus in the literature that provides recommendations on the required number of readings to predict the presence of variability.^9^ In our study, we believe that a minimum of 10 readings over the course of the first 3 days of stroke onset can be a good predictor of variability. Hence, our study shows that additional research is necessary to find parameters that may help indicate patients in greater risk for a higher BPV following a cerebrovascular accident. Furthermore, since our study focuses mainly on one outcome measure, which is mortality, more well-controlled studies need to be initiated to meticulously investigate BPV effects on other outcomes, including functional and neurologic outcomes. Finally, finding potential effective therapy for increases in SBPV and DBPV is crucial to optimise the therapeutic approach in stroke patients to minimise the negative effects of BPV. There are several emerging studies comparing the effect of different pharmacological agents on BPV after an ischaemic stroke. Drugs like fimasartan have shown these effects in a study conducted by Shin *et al*.^42^ In a recently published narrative review, they discuss that the use of antihypertensive medications like calcium channel blockers and non-loop diuretics was found to reduce BPV in outpatient visit-to-visit BP readings when used alone or in combination with other agents.^43^ In contrast, beta blockers were observed to increase BPV in similar settings, which can be considered when starting inpatient antihypertensive medications for BP control. Additionally, it was recommended to avoid the use of potent short-acting medications as it would exacerbate the iatrogenic potential of such action on BPV.^43^

Our study has several points of strength. It is noteworthy that we included a cohort of 2572 patients who represent a relatively young population with a male sex majority due to the make-up of the country, which has been reported previously and is multiethnic with majority from MENA and South Asia. This makes the dataset robust and allows for generalisability of our results. The results also show strong evidence against the null hypothesis, giving us a valuable insight that is clinically relevant to the association between different BPV parameters and stroke-related mortality. The statistical analysis techniques employed showed robust good results with ROC curves, which helps us assess the predictability of mortality based on parameters like SBPV and DBPV. Additionally, baseline characteristics and confounders were well-controlled and reported. Finally, we have also had a prolonged follow-up period of up to 1 year after stroke onset in which mortality has been reported. In addition, we assessed the causal impact of BPV on mortality by adjusting for potential confounders determined through a DAG. In addition, we were able to demonstrate a dose–response relationship between the level of BPV and mortality, further strengthening the causal evidence of our results.

### Limitations

Our study is limited by the inevitable consequences usually associated with its retrospective design, including dealing with missing values, and the absence of consistent synchrony in the timing of BP measurements by nursing personnel. Our results could also be affected by the inability to adjust for factors that may adversely affect, BP like physical activity, caffeine intake, smoking, among others. Nevertheless, BP was consistently measured while patients are supine and through arm measurement, an appropriate cuff was used and other factors like adequate rest prior to measurements were considered. Additionally, the stability of point estimates of our study outcomes meant that these limitations did not significantly confound our findings.

## Conclusion

In a retrospective cohort of ethnically diverse acute stroke patient population, BPV was significantly associated with both in-hospital and 1-year mortality. Clinical outcomes beyond 1 year remain uncertain. There is a need to explore these findings further through a prospective examination of large patient cohorts to both ascertain the validity of these findings, as well as provide a prescriptive direction on BP management in acute stroke management.

## Supplementary material

10.1136/bmjopen-2024-095773 online supplemental file 1

## Data Availability

Data are available upon reasonable request.
