# Supplementary material for: Blood pressure variability and mortality in patients admitted with acute stroke in a tertiary care stroke centre (2016–2019): a retrospective cohort study
Source: BMJ Open. 2025 May 15;15(5):e095773. doi: 10.1136/bmjopen-2024-095773 (PMC12083430; doi:10.1136/bmjopen-2024-095773)
Supplement: online supplemental file 1 [file bmjopen-15-5-s001.docx]

Table 1. Baseline characteristics of the patients.

| Variable | Level | Total  (n=2,554) | Alive  (n=2,417) | Dead  (n=137) | P-Value |
| --- | --- | --- | --- | --- | --- |
| **Age, years** | Median (IQR) | 53 (44-62) | 53 (44-61) | 55 (42,68) | 0.19 |
| **Sex** | n (%) |  | | | 0.036 |
| Female |  | 446 (17.5%) | 413 (17.1%) | 33 (24.1%) |  |
| Male |  | 2,108 (82.5%) | 2,004 (82.9%) | 104 (75.9%) |  |
| **Ethnicity** | n (%) |  | | | 0.33 |
| MENA |  | 893 (35%) | 844 (34.9%) | 49 (35.8%) |  |
| South Asia |  | 1,302 (51.0%) | 1,240 (51.3%) | 62 (45.3%) |  |
| Southeast Asia |  | 258 (10.1%) | 238 (9.9%) | 20 (14.6%) |  |
| East Asia |  | 9 (0.4%) | 9 (0.4%) | 0 (0.0%) |  |
| Others |  | 92 (3.6%) | 86 (3.6%) | 6 (4.4%) |  |
| **BMI** | Median (IQR) | 27.1 (24.3-30.4) | 27.18 (24.4-30.4) | 26.3 (24.2-30) | 0.39 |
| **Admission NIHSS** | Median (IQR) | 3 (2-8) | 3 (2-7) | 18 (7-25) | <0.001 |
| **Diagnosis** | n (%) |  | | | <0.001 |
| ICH |  | 421 (16.5%) | 355 (14.7%) | 66 (48.2%) |  |
| Ischemic Stroke |  | 2,133 (83.5%) | 2,062 (85.3%) | 71 (51.8%) |  |
| **Smoking Status** | n (%) |  | | | <0.001 |
| Non-Smoker |  | 1,839 (72.0%) | 1,717 (71.0%) | 122 (89.1%) |  |
| Smoker |  | 585 (22.9%) | 574 (23.8%) | 11 (8.0%) |  |
| Ex-Smoker |  | 128 (5%) | 124 (5.1%) | 4 (2.9%) |  |
| Tobacco Chewer |  | 2 (0.1%) | 2 (0.1%) | 0 (0%) |  |
| **History of Cardiac Disease** |  |  | | |  |
| Yes | n (%) | 379 (14.8%) | 343 (14.2%) | 36 (26.3%) | <0.001 |
| Not Known |  | 2,175 (85.2%) | 2,074 (85.8%) | 101 (73.7%) |  |
| **Hypertension** | n (%) |  | | | <0.001 |
| Not known |  | 624 (24.4%) | 578 (23.9%) | 46 (33.6%) |  |
| Known |  | 1,458 (57.1%) | 1,375 (56.9%) | 83 (60.6%) |  |
| Newly Diagnosed |  | 471 (18.5%) | 463 (19.2%) | 8 (5.8%) |  |
| **Diabetes** | n (%) |  | | | <0.001 |
| Not Known |  | 867 (34%) | 801 (33.1%) | 66 (48.2%) |  |
| Known |  | 1,039 (40.7%) | 987 (40.8%) | 52 (38%) |  |
| Newly Diagnosed |  | 241 (9.4%) | 232 (9.6%) | 9 (6.6%) |  |
| Pre-diabetes |  | 407 (15.9%) | 397 (16.4%) | 10 (7.3%) |  |
| **Hyperlipidemia** | n (%) |  | | | <0.001 |
| Not Known |  | 1,305 (51.1%) | 1,209 (50.0%) | 96 (70.1%) |  |
| Known |  | 461 (18.1%) | 433 (17.9%) | 28 (20.4%) |  |
| Newly Diagnosed |  | 788 (30.9%) | 775 (32%) | 13 (9.5%) |  |
| **History of Stroke** | n (%) |  | | | 0.81 |
| Not Known |  | 2,310 (90.5%) | 2,184 (90.4%) | 126 (92%) |  |
| Known |  | 244 (9.6%) | 233 (9.6%) | 11 (8%) |  |
| **History of TIA** | n (%) |  | | | 0.92 |
| Not Known |  | 2,537 (99.3%) | 2,401 (99.3%) | 136 (99.3%) |  |
| Known |  | 17 (0.7%) | 16 (0.7%) | 1 (0.7%) |  |
| **History of DVT** | n (%) |  | | | <0.001 |
| Not Known |  | 2,545 (99.7%) | 2,411 (99.8%) | 134 (97.8%) |  |
| Known |  | 9 (0.4%) | 6 (0.3%) | 3 (2.2%) |  |
| **Admission Labs** |  | | | | |
| HbA1C | Median (IQR) | 6.2 (5.5-8.6) | 6.2 (5.5-8.6) | 6.7 (5.8-10.4) | 0.040 |
| Cholesterol | Median (IQR) | 4.8 (4-5.7) | 4.8 (4-5.7) | 4.2 (3.7-5.3) | 0.087 |
| Triglycerides | Median (IQR) | 1.4 (1-2) | 1.4 (1-2) | 1.3 (1-1.6) | 0.061 |
| HDL | Median (IQR) | 0.97 (0.8-1.2) | 0.97 (0.8-1.2) | 1 (0.9-1.3) | 0.12 |
| LDL | Median (IQR) | 3 (2.3-3.8) | 3 (2.3-3.8) | 2.8 (2-3.7) | 0.13 |
| Platelet | Median (IQR) | 255 (213-306) | 255 (213-306.5) | 249 (214-294) | 0.36 |
| PTT | Median (IQR) | 10.4 (8.7-11.3) | 10.4 (8.7-11.3) | 11 (9.1-12) | <0.001 |
| INR | Median (IQR) | 1 (1-1.1) | 1 (1-1.1) | 1.1 (1-1.2) | <0.001 |
| APTT | Median (IQR) | 26.5 (24.5-29) | 26.5 (24.6-29) | 25.9 (23.4-29.2) | 0.21 |
