# Supplementary material for: Blood pressure variability and mortality in patients admitted with acute stroke in a tertiary care stroke centre (2016–2019): a retrospective cohort study
Source: BMJ Open. 2025 May 15;15(5):e095773. doi: 10.1136/bmjopen-2024-095773 (PMC12083430; doi:10.1136/bmjopen-2024-095773)
Supplement: Supplementary file 2 [file bmjopen-2024-095773.reviewer_comments.pdf]

PEER REVIEW HISTORY

BMJ Open publishes all reviews undertaken for accepted manuscripts. Reviewers are asked to complete a checklist review form and are provided with free text boxes to elaborate on their assessment. These free text comments are reproduced below.

ARTICLE DETAILS

Title (Provisional)

Blood Pressure Variability and Mortality in Patients Admitted with Acute Stroke in a Tertiary Care Stroke Center (2016-2019); A Retrospective Cohort Study

Authors

Tawengi, Mohamed; Hourani, Rizeq F.; Alyaarabi, Tamader; Elsabagh, Ahmed Adel; Al-Dali, Yazan; Ghassan Hommos, Rama; Baraka , Jawaher; Tawengi, Abdelaziz M; Abdallah, Bushra M; Hatem, Ahmad; Sardar, Sundus; Imam, Yahia Z.; Akhtar, Naveed; Zahid, Muhammad; Doi, Suhail; Danjuma , Mohammed Ibn-mas'ud; Elzouki, AbdelNaser

---

VERSION 1 - REVIEW

**Reviewer**	**1**
**Name**	**Jonsdottir, Gudrun**
**Affiliation**	**Faculty of Nursing and Midwifery, School of Health Sciences, University of Iceland**
**Date**	**10-Dec-2024**
**COI**	**None**

It is an interesting study. There is a clear statement of aim and purpose outlining the study's objectives.

The retrospective quantitative study design is appropriate for achieving the stated objectives.

Including an ethics statement in the paper itself would strengthen transparency and accountability. Additionally, an explanation regarding the absence of informed consent from patients would have been beneficial to address potential ethical concerns.

The paper needs to address data protection measures sufficiently. Details regarding how patient data was secured and privacy maintained would be crucial to adhere to ethical standards.

A potential limitation of the study lies in the need for more control over factors that could influence blood pressure measurements, such as recent physical activity (e.g., standing or

moving) or inconsistencies in the selection of the arm for measurement. These factors could affect variability in the results and should be discussed.

The references cited in the paper are somewhat dated. It would be good to confirm whether a comprehensive literature search was conducted to include more recent and relevant studies.

**Reviewer**	**2**
**Name**	**Moullaali, Tom**
**Affiliation**	**University of Edinburgh, Centre for Clinical Brain Sciences**
**Date**	**15-Jan-2025**
**COI**	**None**

Thanks for asking me to review this retrospective study assessing the association of systolic blood pressure variability (measured by standard deviation of up to 18 measures over 3 days) after acute ischaemic stroke and outcome in a hospital-based cohort from Qatar.

The authors report an association between higher levels of BP variability and poor outcomes after ischaemic stroke.

The main reservation I have is that Manning and colleagues published a systematic review showing similar findings 10 years ago. Other studies have emerged since and most corroborate the association. Other than including people from North Africa (previous studies involved people mainly for Europe, America, China), I think there is little new here.

In terms of methods, the retrospective design and missing data (BP and follow-up) are major limitations. Consideration has not been made to accounting for mean BP (strongly correlated with SD of BP, and higher mean BP levels are known to be associated with poor outcomes after stroke) or testing consistency across other BP variability metrics.

VERSION 1 - AUTHOR RESPONSE

Reviewer 1 Comments:

Comment 1:

Including an ethics statement in the paper itself would strengthen transparency and accountability. Additionally, an explanation regarding the absence of informed consent from patients would have been beneficial to address potential ethical concerns. The paper needs to address data protection measures sufficiently. Details regarding how patient data was secured and privacy maintained would be crucial to adhere to ethical standards.

Response:

Thank you for your comment. Please review the revised ethics statement:
This study utilized retrospectively collected data. Patients' identifiers including name and healthcare numbers were removed, and subjects were anonymized by numbers. The data was stored into an Excel sheet which was secured by a password. It was then shifted to analysis which was also protected by a passcode. Given the retrospective design and the inability to trace data back to individual subjects, informed consent was not required and was waived by the Medical Research Center (MRC) at HMC. Ethical approval was provided by the MRC at HMC, MRC-01-20-968.

Comment 2:
A potential limitation of the study lies in the need for more control over factors that could influence blood pressure measurements, such as recent physical activity (e.g., standing or moving) or inconsistencies in the selection of the arm for measurement. These factors could affect variability in the results and should be discussed.

Response:
Thank you for your comment. Please review the revised limitations statement:
Our study is limited by the inevitable consequences associated usually associated with its retrospective design, including dealing with missing values, and absence of consistent synchrony in the timing of blood pressure measurements by nursing personnel. Our results could also be affected by inability to adjust for factors that may adversely affect blood pressure like physical activity, caffeine intake, smoking, amongst others. Nevertheless, blood pressure was consistently measured while patients are supine and through arm measurement, appropriate cuff was used, and other factors like adequate rest prior to measurements were considered. Additionally, the stability of point estimates of our study outcomes meant that these limitations did not significantly confound our findings.

Comment 3:
The references cited in the paper are somewhat dated. It would be good to confirm whether a comprehensive literature search was conducted to include more recent and relevant studies.

Response:
Thank you for your comment. More recent and relevant studied have been added. Please review the edited manuscript.

Reviewer 2 Comments:

Comment 1:
The main reservation I have is that Manning and colleagues published a systematic review showing similar findings 10 years ago. Other studies have emerged since and most corroborate the association. Other than including people from North Africa

(previous studies involved people mainly for Europe, America, China), I think there is little new here.

Response:
Thank you for your comment. Our project suggests that although other studies were done, there is still no consensus about how much variability is acceptable, how to monitor blood pressure variability, how to treat it and what medications to use for treatment. Therefore, our project provides solid evidence on the hypothesis that blood pressure variability plays a crucial role in the outcomes of stroke, and we recommend further prospective studies to determine these factors. Also, within the subsets of patients, more information and evidence are needed, including for example patients who underwent thrombolysis/thrombectomy vs. patients who did not.

Comment 2:
In terms of methods, the retrospective design and missing data (BP and follow-up) are major limitations.

Response:
Thank you for your comment. We state in our limitations that the limitations of a retrospective design are inevitable. However, we believe that we applied robust interventions to minimize their effects. Please review the edited limitations paragraph. Additionally, regarding the loss to follow-up, we applied a valid statistical approach of inverse probability weighting which is clearly explained in the statistical analysis part. This approach -although not superior to prospective designs that have no loss to follow-up- provides credible and reliable results.

Comment 3:
Consideration has not been made to accounting for mean BP (strongly correlated with SD of BP, and higher mean BP levels are known to be associated with poor outcomes after stroke) or testing consistency across other BP variability metrics.

Response:
In a recent review on blood pressure variability in acute stroke (reference below), there is no consensus yet on the proper way of defining blood pressure variability. There are different indices being used, of which the standard deviation is a commonly used and a reliable measurement. Additionally, we have calculated the flux which provides further insight on the effect of blood pressure variability within the individual groups, clearly showing that variability carries out a negative impact in hypertensive and hypotensive patients.
Our results are consistent in all analyses that was carried out, and with our large sample size, we believe that we provide solid evidence in the different extremes of blood pressures (represented by the flux -p50-).

Reference: Zompola, C., Palaiodimou, L., Voumvourakis, K., Stefanis, L., Katsanos, A. H., Sandset, E. C., Boviatsis, E., & Tsivgoulis, G. (2024). Blood Pressure Variability in Acute Stroke: A Narrative Review. *Journal of Clinical Medicine*, 13(7), 1981. https://doi.org/10.3390/jcm13071981

Additional Edits:

We noticed a minor error in the total number of patients reported which was corrected accordingly. Edits can be found in the tracked document.

VERSION 2 - REVIEW

**Reviewer**	**2**
**Name**	**Moullaali, Tom**
**Affiliation**	**University of Edinburgh, Centre for Clinical Brain Sciences**
**Date**	**19-Mar-2025**
**COI**	

Thank you for your responses to my comments.

I have not been persuaded that the study adequately answers the core research question: we have known for many years that retrospective analysis of BP variability is associated with poor outcomes after stroke and in other cardiovascular diseases, and that prospective studies aimed at accurately quantifying BP variability and confounders of outcome are needed. The current study does not provide new information in this regard.
